# Exploring the burden, prevalence and associated factors of chronic musculoskeletal pain in migrants from North Africa and Middle East living in Europe: a scoping review

**DOI:** 10.1186/s12889-023-17542-2

**Published:** 2024-03-12

**Authors:** Maria-Nefeli Tsetseri, David J. Keene, Alan J. Silman, Stephanie G. Dakin

**Affiliations:** 1 Nuffield Department of Orthopaedics, Rheumatology and Musculoskeletal Sciences, University of Oxford, B4495, Headington, Oxford OX3 7LD UK; 2 Faculty of Health and Life Sciences, University of Exeter, Exeter, UK

**Keywords:** Chronic, Musculoskeletal, Pain, Immigrants, Migrants, Refugees, Asylum seekers

## Abstract

**Background:**

Immigrants are exposed to numerous risk factors that may contribute to the development of chronic musculoskeletal pain. Recent political and environmental crises in North Africa and the Middle East have led to an increase in immigration to Europe that has challenged the healthcare system and especially the management of chronic conditions.

**Objective:**

The aims of this scoping review are to investigate the burden, prevalence, and associated factors of chronic musculoskeletal pain in immigrants from North Africa and the Middle East in Europe during the last decade. The intentions of the review are to inform healthcare policymakers, to identify gaps in the literature, and aid the planning of future research.

**Design:**

Online databases Medline, Embase, PubMed and Web of Science were used to identify epidemiological studies published from2012–2022 examining chronic pain in populations from North Africa and the Middle East with a migration background residing in Europe.

**Results:**

In total eleven studies were identified conducted in Norway (*n* = 3), Denmark (*n* = 3), Germany (*n* = 1), Austria (*n* = 1), Sweden (*n* = 1), and Switzerland (*n* = 1). Among the identified studies, eight studies were cross-sectional (*n* = 8), two were prospective cohort studies (*n* = 2) and one was a retrospective cohort study (*n* = 1). Data suggested that chronic pain is more prevalent, more widespread, and more severe in people with than without a migration background. Furthermore, immigrants who have resided in the destination country for a longer period experience a higher prevalence of chronic pain compared to those in the early phases of migration. The following factors were found to be associated with chronic pain in this population: female gender, lower education, financial hardship, being underweight or obese, time in transit during migration, experience of trauma, immigration status, anxiety, depression, and post-traumatic stress disorder.

**Conclusion:**

Several gaps in the literature were identified. Research is limited in terms of quantity and quality, does not reflect actual immigration trends, and does not account for immigration factors. Prospective cohort studies with long follow-ups would aid in improving prevention and management of chronic pain in populations with a migration background. In particular, they should reflect actual immigration trajectories, account for immigration factors, and have valid comparison groups in the countries of origin, transit and destination.

**Supplementary Information:**

The online version contains supplementary material available at 10.1186/s12889-023-17542-2.

## Key points

• Chronic pain is more prevalent in populations with a migration background than without.

• Immigrant women are a particularly susceptible group in developing chronic pain.

• Length of stay, socio-economic class and mental health are other associated factors.

• Current research does not reflect the magnitude and geography of the issue.

**Supplementary Information:**

The online version contains supplementary material available at 10.1186/s12889-023-17542-2.

## Background

According to the United Nations High Commissioner for Refugees (UNHCR), at least a hundred million people were forcibly displaced ‘as a result of persecution, conflict, violence, human rights violations and events seriously disturbing public order’ [1]. Several crises, including but not limited to the on-going Syrian civil war and the renewed conflicts in Afghanistan, Iraq, and Libya, have led to an increase of 70.8 million immigrants from 2018 to 2019 [1]. According to the World Migration Report, it was estimated that in 2019 around 38 million non-European migrants lived in Europe—that is an approximate 10% increase since 2015 [2]. This recent immigration wave has challenged healthcare in Europe not only because of the demographic increase in numbers and diversity, but also because there are differences in health between people with and without a migration background. Various risk factors, stemming from both migration and non-immigration sources, contribute to this health disparity. Immigrants are likely to have been exposed to various mental and physical health risk factors, such as violence, insecurity, and poor hygiene. They are also likely to face multiple barriers to accessing healthcare, such as language difficulties, lack of knowledge of the healthcare system and poor cultural competence among healthcare providers. Because of the exposure to numerous health risks and the lack of sufficient healthcare, people with a migration background are at risk of developing chronic pain.

Chronic pain is defined as pain that persists beyond the normal tissue healing time which, in the absence of other factors, is usually three months [3]. Chronic pain affects one in five people globally and is the leading cause of disability worldwide [4, 5]. Emerging evidence suggest that immigrants face a heightened risk for the development and maintenance of chronic pain. For example, studies in the UK show that South Asian ethnicity is a significant predictor of spinal pain [6]. Others show that Indian workers living in the UK are at a higher risk of developing widespread pain than Indian workers without a migration background [7]. While Bangladeshi people with lower acculturation levels experience more pain than both those with higher acculturation levels and Whites as referred to in the original article, of the same neighbourhood [8].Taken together, this data suggests that non-genetic immigration factors may be linked to the emergence and maintenance of chronic pain. The physical and mental health, as well as the integration to the host country, of people with a migration background may further be impacted by the experience of chronic pain.

Despite that understanding and promoting migrant health is of crucial humanitarian, social and economic importance, there is a noticeable gap in the literature regarding the current and on-going migration trends. This review aims to identify these gaps, aid to the planning of future research and inform European healthcare policymakers by investigating the burden, prevalence, and associated factors of chronic musculoskeletal pain in immigrants from North Africa and the Middle East in Europe during the last decade.

## Methodology

Because of the complexity of chronic pain and immigration and the heterogeneity among the studies, a scoping review was conducted [9, 10]. The Preferred Reporting Items for Systematic Reviews and Meta-Analyses for Scoping Reviews (PRISMA-ScR) guidelines were followed [10].

### Protocol and registration

There is no review protocol.

### Eligibility criteria

#### Type of studies

This review includes observational studies that were published in peer-reviewed journals during the last decade (2012–2022) in English, German and Greek. The latter two were included because Germany is the second most common destination for migrants worldwide and because Greece is an important transit country for immigrants from North Africa and the Middle East due to its geographic location [2].

#### Type of participants

This review includes all studies reporting chronic, musculoskeletal, widespread, or unspecified pain in non-European immigrants, migrants, refugees, asylum seekers or foreign-born adults residing in European countries. It focuses on immigrants from the Middle East and North Africa.

Migrants moving to Europe from neighbouring countries experience a distinct context in terms of geography, culture, language, immigration policies, and social and healthcare services. Because of the high complexity of the subject, the scope of the study was narrowed to this specific regional subgroup to allow a targeted examination of migration and health-related factors and to gather more precise data and insights. This focused view can be particularly valuable for European policymakers and healthcare providers in addressing the needs of this population. To maximise the inclusion of the research available, studies with a heterogeneous immigrant sample of many nationalities that include but are not limited to migrants from the Middle East and North Africa were also reviewed.

Immigrants, refugees and asylum-seekers are distinct categories of individuals who have moved, are moving, or are willing to move to a different country. While distinct in terms of their legal status and reasons for migration, they share commonalities in their experience of migration and integration (language barriers, economic challenges, barriers to accessing social services, psychosocial stress, discrimination).

#### Exclusion criteria

Exclusion criteria and the justification for each can be found in Table 1 below.

**Table 1:** Exclusion criteria

Exclusion criteria	Justification
Studies that are not observational	The aim of the review is to examine the burden, prevalence and risk factors of chronic pain in immigrants
Studies published prior to 2012	The aim of the review is to capture the most recent immigration trends, during which migration has climbed to a high record due to complex political and environmental reasons
Studies that focus on acute conditions/pain	The focus of the review is on chronic pain
Studies on malignant, metabolic, infectious, visceral, neuropathic or post-surgical pain	The focus of the review is on musculoskeletal pain
Studies with samples that do not include participants from North Africa and the Middle East	The review focuses on the recent political and immigration crises in the countries neighbouring to Europe
Studies where the destination country is not European	The review aims to inform European healthcare policies

### Search strategy

The database PubMed was initially searched to provide an overview of the literature. Maria-Nefeli Tsetseri screened keywords, titles, and articles in order to conduct a second systematic search using all identified key-words in the following databases: Medline, Embase, PubMed and Web of Science. See Additional file 1 for the detailed search in PubMed. Articles were imported in the reference management software EndNote 20.0.1 and duplicate records were removed. Keywords, titles and abstracts were reviewed and screened by Maria-Nefeli Tsetseri. The reference lists of included papers were manually searched to identify additional studies, and Google Scholar was used to screen articles citing the selected studies.

### Data items and data collection process

The authors extracted and tabulated data. The following items were abstracted:

- Study characteristics
  - ◦ year of publication
  - ◦ country
  - ◦ objectives
  - ◦ study design
- Definitions of immigration and chronic pain when present
- Participant characteristics
  - ◦ sample size
  - ◦ gender ratio
  - ◦ mean age
  - ◦ country of origin
  - ◦ country of destination
  - ◦ migration status
- All chronic/musculoskeletal pain outcomes
  - ◦ prevalence
  - ◦ location
  - ◦ duration
  - ◦ risk factors
  - ◦ associations
  - ◦ treatment
  - ◦ rehabilitation referral
  - ◦ painkiller usage
- Mental health outcomes.

### Synthesis of results

A narrative synthesis was undertaken.

## Quality appraisal

Due to the lack of a universal quality appraisal tool for observational studies of different designs and the high complexity of the subject, a unique quality appraisal approach was developed. This appraisal approach was designed to capture the complexity and the specific epidemiological challenges, and to assess the validity and the reliability of the included studies. The tool was developed based on the Newcastle–Ottawa Scale [11] and adapted to fit the purposes of this review. Studies were assessed on five domains, listed below, and each domain was assigned a score with a range of -1 to 1 (low: -1, adequate: 0, high: 1), it follows that total score ranged from -5 to 5 (poor quality: -5, -4; low quality: -3, -2; moderate quality: -1, 0, 1; high quality: 2, 3; excellent quality: 4, 5). Maria-Nefeli Tsetseri assessed each study and prepared a report with a descriptive evaluation of each domain for all studies. The report was discussed and approved by two other reviewers (David J. Keene and Alan J. Silman) and can be found in Additional file 2.

1. Data collection: how data is collected
2. Representativeness: how generalisable are results
3. Exposure: how immigration is defined, measured, and whether other immigration factors are taken into consideration
4. Outcome: how chronic pain is defined, measured and whether other pain characteristics are taken into consideration
5. Study design: assessment of the strengths and limitations of the study design.

## Results

### Literature search

**Fig. 1:**
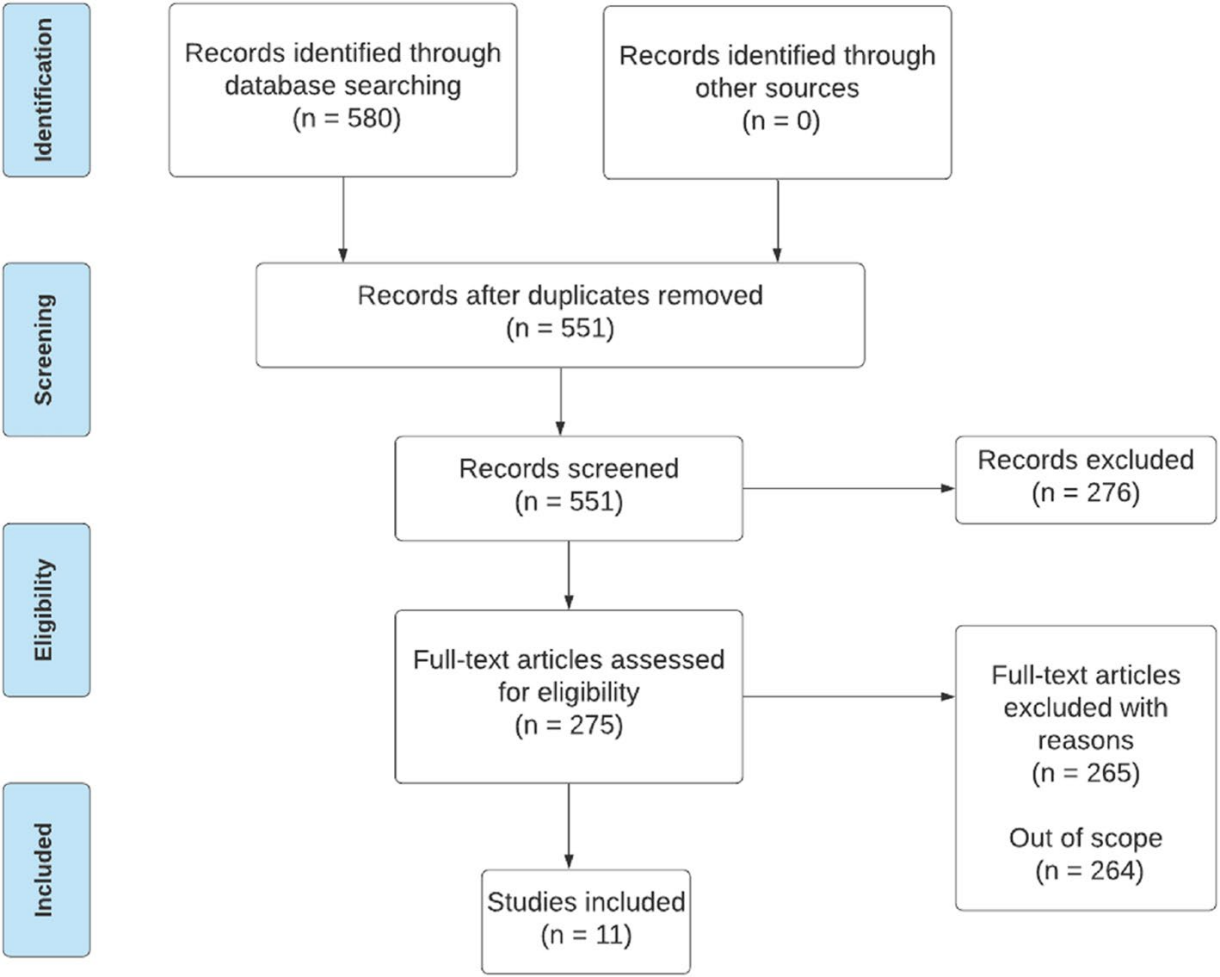
Summary of study selection process

Figure 1 illustrates the identification, screening, and inclusion of studies. The literature search resulted in 580 citations and, after the removal of duplicates, the tittles and abstracts of 551 articles were screened. Of these, 275 full-text articles were assessed; 264 articles were excluded because they were out of scope. Subsequently, 11 studies were included.

### Study characteristics and results

In total eleven studies were identified conducted in Norway (*n* = 3), Denmark (*n* = 3), Germany (*n* = 1), Austria (*n* = 1), Sweden (*n* = 1), and Switzerland (*n* = 1). Eight studies were cross-sectional, two were prospective cohort studies, and one was a retrospective cohort study. One study was published in German and no study published in Greek was found. Additional file 3 detailed the characteristics and the results of each study included in this review.

### Critical appraisal of individual studies

Table 2 presents the summarised quality appraisal; a detailed critical appraisal of each study can be found at Additional file 2.

**Table 2:** Quality appraisal

Reference	Data collection	Representativeness	Exposure: immigration	Outcome: Chronic pain	Study design	Total	Quality
Kesh 2021 [12]	0	-1	-1	-1	-1	**-4**	**poor**
Strømme 2020 [13]	0	0	1	1	1	**3**	**high**
Strømme 2020 [14]	0	0	1	1	0	**2**	**high**
Rosenkrands 2020 [15]	0	-1	-1	0	-1	**-3**	**low**
Dragioti 2020 [16]	0	1	1	1	1	**4**	**excellent**
Waxenegger 2017 [17]	0	1	0	-1	1	**1**	**moderate**
Pfortmueller 2016 [18]	0	1	1	-1	-1	**0**	**moderate**
Führer 2016 [19]	0	0	-1	-1	-1	**-3**	**low**
Teodorescu 2015 [20]	0	-1	0	1	-1	**-1**	**moderate**
Carneiro 2012 [21]	-1	0	0	0	1	**0**	**moderate**
Kurita 2012 [22]	0	1	1	1	1	**4**	**excellent**

Individual domain score: low -1, adequate 0, high 1;

Total score: poor (-5, -4), low (-3, -2), moderate (-1, 0, 1), high (2, 3), excellent (4, 5)

## Synthesis of results

### Prevalence of chronic pain

#### Populations with vs without migration background

Four studies [16, 17, 21, 22] compared pain in populations with a migration background to Swedish [16], Danish [21, 22], and Austrian [17] populations without migration backgrounds. Two studies of excellent quality [16, 22] compared the prevalence of chronic pain, and two studies of moderate quality compared severity [17, 21]. All four studies included clearly defined immigrants as foreign-born. However, there were differences in the definition of chronic pain pertaining to the duration of pain longer than three months [16], longer than six months [22], longer than 30 days [21] and not provided [17] As illustrated in Table 3, the prevalence of chronic pain, of widespread chronic pain, and of severe chronic pain were significantly higher in populations with a migration background compared to populations without. Immigrants also had higher odds of reporting chronic pain, chronic widespread pain and chronic severe pain than individuals without a migration background [16, 22]. Two studies showed that immigrants report higher mean pain intensity and significantly higher total body pain than individuals without a migration background [21, 22]. One study also showed significant differences in musculoskeletal pain intensity between men with and without a migration background^1^ and between women with and without a migration background,^2^ but these differences did not remain significant when adjusting for age, socio-economic status, and health-related behaviours [17]. The only significant difference in musculoskeletal pain intensity that remained was between immigrant women from countries with a low human development index (HDI)^3^ and women without a migration background.

**Table 3:** Chronic pain outcomes: populations with vs without migration background

Outcome	With migration background	Without migration background	*P*- value	Odds ratio	Reference
	**Prevalence**				
Chronic pain	45%	39%	*p* < 0.001	1.18 [1.04–1.33]	[16]
	32.8%	26.4%		1.68 [1.41–1.98]	[22]
Widespread chronic pain	11%	8%	*p* < 0.001	1.39 [1.15–1.69]	[16]
	10.3%	4.6%			[22]
Severe chronic pain	32%	20%	*p* < 0.001	1.51 [1.23–1.87]	[16]
	**Mean pain intensity**				
Mean pain intensity	6.2 out of 10	4.8 out of 10			[22]
	10.1 out of 24	8.2 out of 24	*p* = 0.007		[21]
	0.55^a^ 0.75^b^ 0.65^c^ out of 4 (unadjusted)	0.60 out of 4			[17]

[]: Confidence Interval 95%

^a^from very high HDI

^b^from high HDI

^c^from low HDI

#### Refugees in transit and in early post-migration phase

Three studies investigated the prevalence of chronic pain in immigrants in transit and the early post-migration phase [12–14]. One of the three studies was of poor epidemiological quality and studied victims of torture in a transit country without providing information on the definition of migration nor chronic pain [12]. The other two studies were conducted by the same authors and included Syrian refugees in-transit (in Lebanon) and in the early post-migration phase (in Norway). The latter studies were of high epidemiological quality and measured chronic pain using a standardised, validated tool (> 6 months) [13, 14]. As illustrated in Table 4, the prevalence of chronic pain was estimated at 30% in both the victims of torture in transit [12] and the refugees in transit [14]. While the prevalence of chronic pain in refugees on the early post-migration phase was reported at 37% [14]. This data suggests that chronic pain may be more prevalent in the early post-migration phase ((37–38%) than the transit phase (30%) [19]. However, no significant change in chronic pain was observed in Syrian refugees after a year of resettlement in Norway from Lebanon [13]. It is worth noting that when this data is compared to data on late post-migration cohorts, the prevalence of chronic pain is higher in the later phases of migration by up to 15% than the early ones [16, 22].

**Table 4:** Prevalence of chronic pain in the early phases of migration

**Population**	**prevalence**	**Reference**
Victims of torture in an unnamed country Transit phase	30%	[12]
Syrian refugees in Lebanon Transit phase	30%	[14]
Syrian refugees in Norway Early post-migration phase	37%	[13]

#### Associated factors

##### Demographic and immigration factors

Chronic pain was associated with being female ( [13–15, 20], older age [14, 16, 22], lower education [14, 16, 22], financial hardship [16], and being underweight or obese [22], but not with marital status [16]. Several immigration factors were also associated with chronic pain: time in transit [14], experience of trauma [14], and immigration status [16], but no association was found with migrating alone and not having a residence permit [16].

##### Gender

There are well-established gender differences in the prevalence of chronic pain, and present data confirms that chronic pain is more prevalent in immigrant women than immigrant men in transit and early post-migration, as illustrated in Table 5 [14]. Specifically, Strømme et al. in a cross-sectional study of high epidemiological quality showed that Syrian women in Lebanon (transit phase) exhibited a 7% higher prevalence of chronic pain compared to their male counterparts, while in Norway (early migration phase), Syrian women had an even more significant 17% higher prevalence of chronic pain than Syrian men [14]. They also revealed that female Syrian refugees used more painkillers than immigrant men in both phases [14]. Another cross-sectional study in Norway of moderate quality showed that female refuges with post-traumatic stress disorder (PTSD) had a higher mean of pain locations than male refugees with PTSD [20]. While in Denmark, female immigrants with complex needs had higher prevalences of pain in numerous locations than male immigrants with the same needs [15]., The later study was of poor epidemiological quality and, therefore, the results need to be interpreted with caution. However, it is noteworthy that the sample were 408 ‘immigrants with complex needs’ of which 83% were women [15]. This unbalanced gender ratio may imply that women with migration backgrounds tend to present with more complex symptomatology and multi-morbidity than immigrant men [15]. While a larger Austrian cross-sectional study of adequate epidemiological quality [17] showed that when adjusting for age, socio-economic status and health-related behaviours, significant differences in pain intensity remain between women with and without migration background but not between men with and without a migration background. Even though the intersection of immigration and gender raises concerns about the heightened vulnerability of immigrant women to chronic pain and mental disorders, it is important to note that the highest-quality epidemiological study in this review did not establish that gender has a direct effect on chronic pain [16]. While a small cross-sectional quality of moderate epidemiological quality did not find significant association between chronic pain and no chronic pain for gender among refugees with PTSD [20]. Additional research that adequately represents women with a migration background and considers the nuanced interplays between migration, gender and chronic pain is necessary.

**Table 5:** Gender differences

**Pain symptom reported**	**Female** %,	**Male** %,	**Reference**
Chronic pain	Lebanon: 22% [17–28] Norway: 32% [22–44]	Lebanon: 15% [11–20] Norway: 17% [12–23])	[14]
Painkiller usage	Lebanon: 14% [10–19] Norway: 13% [6–22]	Lebanon: 4% [2–7] Norway: 9% [5–14]	
Pain in arms/hands/legs/knees/hips	87%	83%	[15]
Pain in shoulder/neck	86%	71%	
Pain in upper/lower back	85%	75%	
Headache	83%	75%	
Chronic pain locations	Mean: 5.28 [6.28–4.28]	Mean: 4.08 [6.23–1.95]	[20]

[]: Confidence Interval 95%

##### Mental health

Five studies provided data on mental health (see Table 6). The prevalence of depression, anxiety, and PTSD symptoms in immigrant populations varied from 6 to 55%, 7% to 40% and 7% to 21% respectively, independent of whether participants experienced chronic pain or not. The highest prevalence of depression and anxiety were found in asylum seekers/refugees in transit and in the early stage of post-migration [12, 13, 19], and the lowest prevalence were in the general immigrant populations in Sweden and Denmark. A significant improvement in depression and anxiety of 33% was found from the transit phase to the early post-migration phase [13]. However, depression and anxiety remained significantly more prevalent in immigrants than Swedish people without a migration background [16].

**Table 6:** Mental health in immigrants

Outcome	Prevalence	Population	Quality	Reference
**General mental symptoms**	33.2%	Victims of torture in a transit country	poor	[12]
**Depression**	14.5%	Victims of torture in a transit country	poor	[12]
	18%	Immigrant patients with complex needs in Denmark	low	[15]
	Baseline: 6.5%, follow-up: 6.1%	Foreign-born immigrants in Sweden	excellent	[16]
	54.7%	Asylum seekers in Germany	low	[19]
**Anxiety**	6.9%	Victims of torture in a transit country	poor	[12]
	Baseline: 8.9%, follow-up: 8.4%	Foreign-born immigrants in Sweden	excellent	[16]
	40.2%	Asylum seekers in Germany	low	[19]
**Anxiety/Depression**	35%	Refugees relocating from Lebanon to Norway	high	[14]
**PTSD**	28.9%	Victims of torture in a transit country	poor	[12]
	7%	Refugees relocating from Lebanon to Norway	high	[14]
	19%	Immigrant patients with complex needs	low	[15]
	18.2%	Asylum seekers in Germany	low	[19]

*PTSD* Post-traumatic stress disorder

In refugees with PTSD there were no significant associations between chronic pain and several demographic, immigration, and integration factors^4^ [20]. However, the sample size of that study was small (*n* = 61). The highest percentage of PTSD symptoms was found in victims of torture and in refugees with complex needs (21% and 19% respectively) [12, 15], while patients with chronic pain at clinical levels had significantly more PTSD symptoms and 88% of patients with PTSD had chronic pain at clinical levels [20]. Yet, the difference between those with PTSD and without one in terms of having chronic pain was not significant [20]. These findings show a complex interplay between immigration, history of trauma, mental health and chronic pain.

## Discussion

In total eleven studies were identified conducted in Norway (*n* = 3), Denmark (*n* = 3), Germany (*n* = 1), Austria (*n* = 1), Sweden (*n* = 1), and Switzerland (*n* = 1).Eight studies were cross-sectional, two were prospective cohort studies and one was a retrospective cohort study. The quality of the studies was appraised using a tool developed by the authors of this review to allow the assessment of observational studies of different designs. Two studies were of excellent quality, two of high quality, four of moderate, two of low and, one of poor quality. This review identified four main themes that were associated with chronic pain in populations with a migration background: migration phase (late), gender (female), mental health (anxiety, depression and PTSD), and socioeconomic status.

The findings of this review suggest that chronic pain is more prevalent, more widespread, and more severe in populations with than in populations without a migration background. The high prevalence of chronic pain in immigrants is in line with evidence showing that immigrants report poorer physical health in general [17, 23]. Findings also suggest that chronic pain becomes more prevalent with length of stay in the destination country. Women with a migration background were identified as a particularly vulnerable group for developing chronic pain and comorbid mental health disorders. Apart from gender, older age, lower education, financial hardship, being underweight or obese, time in transit, experience of trauma, and immigration status were also associated with chronic pain in immigrants. Anxiety, depression, and PTSD diagnoses were also shown to be more prevalent in immigrants and were significantly associated with chronic pain.

The provision of healthcare to migrant and refugee populations is hindered by multiple systemic factors. These systemic barriers include legal and administrative complexities, cultural and language differences that lead to communication gaps between healthcare providers and migrant patients, structural racism and inadequate knowledge of healthcare professionals to deal with the unique health needs of populations with a migration background [24, 25]. Additionally, migrants and refugees may be unfamiliar with or lack confidence in the healthcare system making it difficult to navigate the healthcare landscape [24]. These challenges intersect with specific vulnerabilities, such as gender-based discrimination and socioeconomic disadvantage, leading to inadequate engagement with health services and risk of remaining untreated, which may result in chronicity of symptoms. Qualitative research shows that immigrants often feel ignored, disregarded, and not taken seriously during their medical encounters, leading to a sense of isolation, discrimination and stress that further exacerbates pain symptoms [26–28]. At the same time, health professionals feel ill-equipped to deal with ethnic minorities and chronic pain patients [29]. It is therefore essential to develop policies that facilitate the provision of healthcare to patients with a migration background, as well as to develop strategies that inform and educate healthcare professionals and immigrants to eliminate these barriers.

A contributing factor to the high prevalence of chronic pain in populations with migration background is the high prevalence of depression, anxiety and PTSD in immigrant communities. According to the World Health Organisation, the prevalence of anxiety and depression in the overall European population is 25% [30]. Findings of this review indicate a higher prevalence in refugees in transit and asylum seekers (33%, 55% and 40%) [12, 13, 19]. While the study of the highest quality included in this review report a statistically significant higher prevalence of depression and anxiety in the migrant population when comparted to the Swedish-born population [16]. Taking it together present data suggest higher rates of anxiety and depression in populations with than without a migration background during all phases of migration which is in accordance with previous research [31–33]. Review findings also align with expectations, revealing higher rates of PTSD in populations with migration background. The prevalence of PTSD is notably higher in victims of torture (29%), asylum seekers (18%), immigrants with complex needs (19%) and refugees in transit (7%) compared to the general European population, where rates range from 0.56% to 6.67% [12, 13, 15, 19, 34]. These findings are important because they shed light on potential challenges faced by immigrants, such as acculturation stress, discrimination, and healthcare disparities, and underscore the substantial impact of migration-related experience on mental health.

The comorbidity and bidirectionality between mental health disorders and chronic pain is well-established. Current data indicates that this bidirectional relationship between mental health disorders and chronic pain also applies to populations with a migration background. [16, 20]. A large population-based study of excellent quality in Sweden shows that depression and anxiety has a significantly increased effect on chronic pain [16]. While a small Norwegian cross-sectional study of moderate quality including refugee patients with PTSD shows that 88% them experience chronic pain at clinical levels [20]. However, the latter result was not significant because of the small sample of the study [20]. There is a dynamic and reciprocal relationship between mental disorders and chronic pain, known as mutual maintenance mechanism, where each condition contributes to the maintenance and exacerbation of the other. Several biological, psychosocial, and behavioural mechanisms, including central sensitisation and fear-avoidance behaviour, play a pivotal role in the mutual maintenance of chronic pain and mental health disorders. This intricate interplay is further complicated by the intersection with migration-related factors, such as a history of trauma, insecurity, and isolation. These migration-related elements can amplify the complexity of the relationship, influencing the onset, exacerbation, and management of both chronic pain and mental health issues. Recognising and addressing these multifaceted interactions is crucial for developing comprehensive culturally-sensitive multidisciplinary [20, 35].

Chronic pain is not exclusive to immigrants; rather, it emerges from a complex interplay of socio-economic, mental, and lifestyle factors that may be intertwined with immigration. Chronic pain in immigrants is shown to be associated with lower socio-economic status, reduced educational attainment, high and low body mass index, and compromised mental health. The observed prevalence of chronic pain is potentially better explained by the impact of lower socio-economic and education statuses, which hinder health-promoting behaviours such as exercising, better diet, getting regular treatments, and social engagement [36, 37]. Waxenegger et al. [17], demonstrate that differences in physical and psychological quality of life, subjective health, and musculoskeletal pain between individuals with and without a migration background diminish upon adjustment for age, socio-economic status, and health-related behaviours. This highlights the influence of non-immigration factors in shaping physical and mental health, emphasising the need to consider a broader array of contextual variables in understanding health outcomes in populations with a migration background. However, significant differences in physical quality of life, subjective health and musculoskeletal pain between women without a migration background and immigrant women from countries with low HDI remain after adjustment, which suggests that gender and pre-immigration factors are important determinants of physical health. A low HDI may indicate gender-disparities in accessing education and healthcare. This data suggests that women with migration backgrounds from countries with low HDI may continue to face barriers accessing social services in the host country that needs to be taken into account by policymakers aiming at reducing gender inequalities and improving migrants’ well-being.

The present data shows that there is no change in the prevalence of chronic pain from the transit to the early post-migration phase. However, the prevalence of chronic pain is higher in the general immigrant population than in early immigrants, suggesting that chronic pain is higher in the late post-migration phase. This is in line with the view that migrant health deteriorates with length of stay, known as ‘migrant exhausted effect’ theory [13, 38, 39]. Qualitative research on immigrants with chronic pain provides important insights into how unfulfilled expectations and mental exhaustion may be linked to the development of chronic pain [27]. Several factors, such as unemployment and insecurity, loss of socio-economic status, collision of norms, and comparison to life at home lead to feelings of hopelessness, loneliness, and loss of self that both impact and cause pain [27, 40]. Mixed-method longitudinal research would allow for further investigation of the complex biopsychosocial factors linking migration and chronic pain.

Previous research reports higher prevalence of chronic pain and of depression comorbid with chronic pain in women, which is in line with present data on immigrant women compared to immigrant men [4, 41, 42]. Biological differences, gender roles, and cognitive factors have been proposed to explain the observed differences in the prevalence of chronic pain, but the issue remains inconclusive [42]. Various studies report cultural and gender differences in the perception, expression, and tolerance of pain, while negative medical encounters are found in both immigrant and female patients [28, 42]. To the authors’ knowledge, there are no studies comparing pain thresholds, coping mechanisms, and medical encounters between women with and without a migration background, nor between immigrant women and immigrant men. Nevertheless, immigrant women in pain find themselves in the intersecting structure of ethnicity, class and gender that leads to multiple forms of isolation: at home, at work, socially and in clinical settings [26, 43]. This may explain why immigrant women with chronic pain are more likely to be on longer sick leave than women without a migration background, to have depression, to use more painkillers, to experience more severe pain, and to have more pain sites than immigrant men [44].

### Limitations

Available data and research do not reflect the magnitude nor the geographical distribution of immigrants in Europe. Only eleven studies were identified, seven of which were conducted in Scandinavia. Though most immigrants from North Africa and the Middle East enter Europe by crossing the Mediterranean Sea, no studies conducted in Spain, Italy or Greece were found. Only one study in Spain was identified, focusing on the prehospital care, but it investigated acute conditions and was therefore not included in this review [45]. Germany is the second highest destination for migrants worldwide, and the United Kingdom, France and Italy are among the ten highest destination countries [2]. It is therefore surprising that only one study in Germany was found, and none in the United Kingdom, France, or Italy. Two studies that analysed data on immigrant health from outpatient clinics in Germany were identified but were not included because they did not investigate chronic pain. Both reported high rates of musculoskeletal symptoms [46, 47].

One of the major challenges to this review was the lack of any single universal definition for either the exposure (immigration) or the outcome (chronic musculoskeletal pain). The methodology and samples of the included studies were heterogeneous within and among the studies—in terms of demographic and immigration characteristics—which raises the question of comparability of results. Studies with different definitions of chronic pain were included, and most did not categorise chronic pain as musculoskeletal. In most studies, the outcome was self-reported using methods that were not translated or culturally validated. These factors not only make the comparability and synthesis of results challenging, but also raise questions of the relevance and validity of findings. Very few studies investigate the impact of migration factors and acculturation, and even fewer compare findings to populations without a migration background. In fact, no study compared findings to the population of the country of origin that did not migrate. The challenges and concerns regarding validity are accentuated by the utilisation of non-validated appraisal tool.

## Conclusion

Migration waves during the last decade have challenged European policymakers and healthcare systems, as the health needs of immigrants tend to differ from those without a migration background, influenced by a complex interplay of migration and non-migration factors. This review showed that chronic pain is more prevalent in immigrants and is associated with various factors, including longer length of stay at the destination country, being female, low socio-economic status, and comorbid mental health disorders.

Several gaps in the literature were identified. Research is limited in terms of quantity and quality, does not reflect actual immigration trends, and does not account for immigration factors. Prospective cohort studies with long follow-ups would aid in improving prevention and management of chronic pain in populations with a migration background. These studies should depict actual immigration trajectories, account for immigration and non-immigration factors, and include valid comparison groups in the countries of origin, transit and destination.

For future treatment and research, policies should focus on dismantling systemic barriers, facilitating access to healthcare services, educating healthcare professionals and informing patients with a migration background. Robust epidemiological studies are crucial for informing policymakers and enhancing strategies to prevent and manage chronic pain in immigrant populations.

### Supplementary Information

**Additional file 1.** Detailed PubMed search.

**Additional file 2.** Quality appraisal of individual studies.

**Additional file 3.** Study characteristics and results.

## Data Availability

The dataset of this study is available from the corresponding author upon request.

## Footnotes

1 From countries of very high and low HDI.

2 From countries of very high and low HDI.

3 HDI is an important tool for measuring human well-being and addressing gender inequalities as it takes into account various dimensions beyond just economic factors, such as health and education.

4 Demographic, immigration and integration factors: age, education, marital status, employment, ethnicity, living conditions, Norwegian language proficiency, integration in Norway and in ethnic community, and years living in Norway.
