# Supplementary material for: Exploring the burden, prevalence and associated factors of chronic musculoskeletal pain in migrants from North Africa and Middle East living in Europe: a scoping review
Source: BMC Public Health. 2024 Mar 12;24:769. doi: 10.1186/s12889-023-17542-2 (PMC10935970; doi:10.1186/s12889-023-17542-2)
Supplement: Supplementary file 1 — Additional file 1. Detailed PubMed search. [file 12889_2023_17542_MOESM1_ESM.docx]

**Additional file 1. Detailed PubMed search**

((((Pain*[Title/Abstract] OR Ache*[Title/Abstract]) OR ("Pain"[Mesh])) AND ((chronic*[Title/Abstract] OR myalgia[Title/Abstract] OR musculoskeletal[Title/Abstract] OR muscle[Title/Abstract] OR arthralgia[Title/Abstract] OR joint*[Title/Abstract] OR neck*[Title/Abstract] OR back*[Title/Abstract] OR widespread[Title/Abstract]) OR ((("Musculoskeletal Pain"[Mesh])) OR "Chronic Pain"[Mesh]))) AND (((((("Refugees"[Mesh])) OR "Emigration and Immigration"[Mesh]) OR "Transients and Migrants"[Mesh]) OR "Emigrants and Immigrants"[Mesh]) OR (refugee*[Title/Abstract] OR migrant*[Title/Abstract] OR immigrant*[Title/Abstract] OR asylum[Title/Abstract] OR displaced[Title/Abstract] OR diaspora[Title/Abstract] OR emigrant*[Title/Abstract]))) AND ((prevalen*[Title/Abstract] OR inciden*[Title/Abstract] OR frequen*[Title/Abstract] OR rate[Title/Abstract] OR epidemiolog*[Title/Abstract] OR demograph*[Title/Abstract] OR occur*[Title/Abstract]) OR (((("Epidemiology"[Mesh:NoExp]) OR "Prevalence"[Mesh]) OR "Demography"[Mesh]) OR "Incidence"[Mesh] OR “Risk Factors”[Mesh] OR “Risk Factor*[Title/Abstract] OR “Outcome*[Title/Abstract]))
