# Supplementary material for: Exploring the burden, prevalence and associated factors of chronic musculoskeletal pain in migrants from North Africa and Middle East living in Europe: a scoping review
Source: BMC Public Health. 2024 Mar 12;24:769. doi: 10.1186/s12889-023-17542-2 (PMC10935970; doi:10.1186/s12889-023-17542-2)
Supplement: Supplementary file 2 — Additional file 2. Quality appraisal of individual studies. [file 12889_2023_17542_MOESM2_ESM.docx]

**Additional file 3. Quality appraisal of individual studies**

Studies were assessed on five domains, that are listed below, and each domain was assigned a score with a range of -1 to 1 (low: -1, adequate: 0, high: 1).

1. **Data collection:** how data is collected
2. **Representativeness:** how generalisable results are
3. **Exposure:** how immigration is defined, measured and whether other immigration factors are taken into consideration
4. **Outcome:** how chronic pain is defined, measured and whether other pain characteristics are taken into consideration
5. **Study design:** the strengths and limitations of the study design are assessed.

**Quality appraisal of individual studies**

| Study | |
| --- | --- |
| Offering care for victims of torture among a migrant population in a transit country: a descriptive study in a dedicated clinic from January 2017 to June 2019 (Keshk et al 2021) – Total score: -4 | |
| 1. Data collection – Score: 0 | |
| This descriptive retrospective cohort study is important because it analyses routine data collected from a multidisciplinary *Médecins Sans Frontières* clinic. Analysing clinical data minimises recall bias but may suffer from bias associated with healthcare seeking. | |
| 1. Representativeness – Score: -1 | |
| Due to confidentiality and safety reasons, key information is not reported and analysed, including where the study is conducted or demographic and immigration characteristics of the participants, which severely limits the generalisability of the study.  The sample size is relatively large (n = 2,512); however, all patients are victims of torture, which reduces the generalisability of findings since nearly all survivors of torture have chronic disorders (64). The balanced gender ratio represents the general population, however immigrant populations, especially victims of torture, tend to have higher male representation. Most participants are between 20 to 45 years old, reflecting the younger age of refugee populations, but not of the general population. The sample is, therefore, partially representative of the immigrant population. | |
| 1. Exposure: immigration – Score: -1 | |
| No information is provided on how immigration is defined and reported. Other immigration factors are not reported, analysed or controlled for. | |
| 1. Outcome: chronic pain – Score: -1 | |
| No information is provided on how chronic pain is defined, assessed or reported. No further pain characteristics are reported, analysed or controlled for. | |
| 1. Study design — Score: -1 | |
| An advantage of the study is the two-year follow-up.  Loss-to-follow-up is very high in all four departments of the study (medical: 60.1%, mental health: 68.2%, physiotherapy: 58%, social: 79.1%) which poses a serious threat to the validity of the study. A strategy to prevent loss-to-follow-up and minimise attrition bias would have potentially increased the rigour of the study. Comparing outcomes to an unexposed group would also have strengthened the study. | |
| Study | |
| Changes in health among Syrian refugees along their migration trajectories from Lebanon to Norway: a prospective cohort study (Strømme et al 2020) – Total score: 3 | |
| 1. Data collection – Score: 0 | |
| This is a prospective cohort study that assesses changes in health from the transit phase to one year into the post-migration phase. At baseline, symptoms are self-reported via a questionnaire in Arabic while, at follow-up, data are collected via a structured phone interview in Arabic. Data are, therefore, not verified by clinical assessment and may be subjected to recall bias. The change of mode of data collection may optimise response rates, however, it poses the question of outcome comparability from baseline to follow-up. | |
| 1. Representativeness – Score: 0 | |
| Data from 353 Syrians translocating from Lebanon to Norway are analysed. The sample size is relatively small, and homogeneous in terms of country of origin, transit and destination. The balanced gender ratio reflects the general population, while the younger age of the sample reflects immigrant populations in transit. Fewer traumatic experiences are reported than usual immigrant populations potentially because the participants fled in the first stages of war. The homogeneity of the sample with lower traumatic experiences potentially allows comparability of findings to the native populations in the countries of origin and destination. | |
| 1. Exposure: immigration – Score: 1 | |
| Participants consist of a clearly defined group of Syrian refugees under protection by the UNHCR in Lebanon resettling to Norway. Various immigration factors are reported and accounted for—length of stay in Lebanon, stay in other transit countries, residence permit, solo migration, and exposure to traumatic events—which strengthens and enriches the findings of the study. | |
| 1. Outcome: chronic pain – Score: 1 | |
| An advantage of the study is that chronic pain was clearly defined as experiencing physical pain for at least six months and measured as a single item in a standardized and validated method. Among the studies included in this review, six months is the longest duration used to define chronic pain and potentially more reliable in excluding acute conditions. However, clinical assessment would be even more reliable. No other pain characteristics are reported, which limits the study. | |
| 1. Study design – Score: 1 | |
| One of the strengths of the study is its prospective design with one year follow-up. However, longer follow-up would potentially better encapsulate the effects of immigration on chronic pain. No significant differences are found between the cohort and the loss-to-follow-up group that would be indicative of selection bias. Another advantage of the study is that it controlled for age, gender and immigration factors. Comparison to an unexposed group would strengthen the study. | |
| Study | |
| Health status and use of medication and their association with migration related exposures among Syrian refugees in Lebanon and Norway: a cross-sectional study (Strømme et al 2020) – Total score: 2 | |
| 1. Data collection – Score: 0 | |
| This is a cross-sectional study describing and comparing the health status of Syrians in two different migration states: in transit in Lebanon and in the early post-migration phase in Norway. A self-completed questionnaire in Arabic is used that poses the threat of recall bias but is more appropriate for Syrian participants. | |
| 1. Representativeness – Score: 0 | |
| The sample of participants in Norway includes both refugees and asylum seekers that have arrived at Norway via various routes and has an unbalanced gender ratio (F: 27%). It is therefore a heterogeneous group in terms of immigration variables, but the gender ratio reflects the overrepresentation of men commonly found in asylum seekers. The rigour of the study is improved by standardising the prevalence proportions to the demographic patterns of Syrians in Norway. However, due to the demographic differences between the samples (i.e., different gender ratios) in Norway and Lebanon, a direct comparison may not be valid. | |
| 1. Exposure: immigration – Score: 1 | |
| Similar to the previous study. | |
| 1. Outcome: chronic pain – Score: 1 | |
| Similar to the previous study. | |
| 1. Study design – Score: 0 | |
| Sample size is relatively small (827) but with a high response rate. The high response rate together with the use of a questionnaire in Arabic with a validated translation and validated survey items increases the rigour of the study. Another strength is that outcomes were adjusted for various immigration variables and demographic characteristics. Comparing outcomes to an unexposed group would have increased the rigour of the study. | |
| Study | |
| Providing targeted healthcare services for immigrants with complex health needs (Rosenkrands et al 2020) – Total score: -3 | |
| 1. Data collection – Score: 0 | |
| This cross-sectional study describes the demographic and health characteristics of immigrant patients with complex needs. Data are collected via a specific screening questionnaire by the clinic doctor during or after the consultation. This method combines self-reporting with clinical evaluation and potentially decreases recall bias, however, the questionnaire was not culturally validated. | |
| 1. Representativeness – Score: -1 | |
| The sample size is relatively small (408) and not representative of the general nor the immigrant population. The sample consists of immigrants with complex symptomatology, multi-morbidity or polypharmacy, of a disadvantaged background in terms of socio-economic status, education and language proficiency, and it has an unbalanced gender ratio (F: 83%). Complex health needs, disadvantaged background, lower acculturation and female gender are all associated with chronic pain and, therefore, results of the study should be interpreted with caution. In addition, mean age is higher than the rest of the studies included in this review (49 years), which is potentially because the majority of the participants have a residence permit of over twenty years and are not asylum seekers or in the early post-migration phase. | |
| 1. Exposure: immigration – Score: -1 | |
| No information is provided on how migration background is defined and measured. Participants represent 43 nationalities, making the sample heterogeneous, but potentially more representative of the immigrant community in Europe. The sample represents an immigration group of low acculturation, since 74% and 72% required oral and written interpretation respectively, despite the long length of holding a residence permit. Some immigration variables are measured but not accounted for (status, length of stay, history of trauma), which would have enriched the study. | |
| 1. Outcome: chronic pain – Score: 0 | |
| Chronic pain is not reported as a primary outcome and is not defined. The study reports prevalence of pain-related symptoms arising from the musculoskeletal system without specifying how and whether symptoms are reported, clinically examined, measured, and without exploring further pain characteristics such as duration, location and severity. However, this is one of the few studies that specifically refers to musculoskeletal pain and not just chronic pain. | |
| 1. Study design – Score: -1 | |
| Further description on how outcomes are defined and measured, adjusting for demographic and immigrant variables, and having an unexposed comparison group would increase the rigour of the study. | |
| Study | |
| Predictive association between immigration status and chronic pain in the general population: results from the SwePain cohort (Dragioti et al 2020) – Total score: 4 | |
| 1. Data collection – Score: 0 | |
| This prospective cohort study examines the association of immigration with chronic pain, chronic widespread pain, and severe pain with a two-year follow-up. Data are collected via a postal questionnaire; this method is subjected to recall bias and may exclude potential participants who are not proficient in written Swedish. | |
| 1. Representativeness – Score: 1 | |
| The sampling frame is based on the Demographic Swedish Total Population Register (TPR) and the sample size is large (14,903), which increases the generalisability of findings. However, response rate at baseline is low (46%) (at follow-up, 73%) and the proportion of immigrant population is relatively low compared to native (10%). The immigration group consists of a heterogeneous population in terms of demographic characteristics, which is potentially more representative of the immigrant community in Sweden but may pose validity issues in the analysis of the outcomes. | |
| 1. Exposure: immigration – Score: 1 | |
| Immigration is defined and measured as individuals who were not born in Sweden according to information in the TPR and data based on country of birth from the questionnaire. This definition has the advantage of being clear and subject to minimum bias, however, no other immigration or acculturation factors—such as length of stay—were measured and accounted for. | |
| 1. Outcome: chronic pain – Score: 1 | |
| Chronic pain is a primary outcome of this study and, it is clearly defined using a single question: ‘Do you frequently have pain lasting more than three months?’. This definition has the advantage of being clear and straightforward, however, it is subjected to recall bias, it is lacking clinical examination and diagnosis, and it is questionable if three months is an adequate duration to categorise pain as chronic. Further, location of pain is reported using a mannequin and severity using a numeric scale (0–10), which enriches the results of the study. | |
| 1. Study design – Score: 1 | |
| A strength of the study is the prospective design with two-year follow-up and a large sample size. However, two years may not be an adequate duration to investigate chronic pain. Several confounders are explored: age, gender, marital status, education level, financial hardship, anxiety, and depression. Interpretation of the results would be enriched if immigration factors and acculturation are accounted for. The study has an unexposed group (native Swedish) which increases the rigour of the study. | |
| Study | |
| The Impact of Migration Background on Health: Results of the Austrian Health Interview Survey (ATHIS 2014) (Waxenegger et al 2017) – Total score: 1 | |
| Data collection – Score: 0 | |
| This is a cross-sectional study analysing data collected from the Austrian Health Interview Survey (ATHIS 2014) using both telephone interviewing and self-administered questionnaires. This method is subject to recall bias. | |
| Representativeness – Score: 1 | |
| Sample size is adequate (n = 15,771) and is stratified by health region and is, therefore, representative of the Austrian native and immigrant population. However, the proportion of immigrant population is relatively low compared to native (10%). | |
| Exposure: immigration – Score: 0 | |
| Immigration is defined as foreign-born or both parents being foreign-born, which is a clear and straightforward definition. However, no information is provided on how that information is obtained. No other immigration factors are reported, analysed or controlled for. | |
| Outcome: chronic pain – Score: -1 | |
| No information is provided on how musculoskeletal pain was defined and measured, and no further pain characteristics are reported, analysed or controlled for. | |
| Study design – Score: 1 | |
| The strengths of the study are its large, stratified sample, and that it adjusts for age, socio-economic status and health-related behaviours (smoking, alcohol consumption, exercising and diet). This is the only study identified that accounts for health-related behaviours. | |
| Study | |
| Adult Asylum Seekers from the Middle East Including Syria in Central Europe: What Are Their Health Care Problems? (Pfortmueller et al 2016) – Total score: 0 | |
| 1. Data collection – Score: 0 | |
| This is a retrospective descriptive study of patients presenting to an emergency department with an official status of asylum seeker or refugee from the Middle East. Data are extracted from medical records, which is a more reliable method than self-reported questionnaires, but no standardised and systematic medical history is taken and may suffer from bias associated with seeking healthcare. | |
| 1. Representativeness – Score: 1 | |
| Sample size is adequate (n = 880) and homogeneous in terms of countries of origin (Middle East), destination country (Switzerland), and immigration status (refugees and asylum seekers). Mean age (34 years) and gender distribution (male: 71%) is representative to asylum seeker and refugee populations from the Middle East, therefore, findings could potentially be generalised to immigrants for the Middle East. | |
| 1. Exposure: immigration – Score: 1 | |
| Immigration status is based on the official resident status routinely assessed by the hospital administration, which is considered a reliable source. No other immigration characteristics are measured and accounted for, limiting the interpretation of the results. | |
| 1. Outcome: chronic pain – Score: -1 | |
| No information is provided on how chronic pain is defined and measured, possibly because of the retrospective design. In addition, data collected from an emergency department may not be relevant and representative for the investigation of a chronic condition. | |
| 1. Study design – Score: -1 | |
| The study is limited due to its retrospective design, lack of comparison group, and of controlling for demographic and immigrant confounders. | |
| Study | |
| Morbidity of asylum seekers in a medium-sized German city (Führer et al 2016 –Total score: -3 | |
| 1. Data collection – Score: 0 | |
| This cross-sectional study aims at describing physical health among asylum seekers in Germany living in shared accommodation. Physical health is self-reported using a translated version of the Harvard Trauma Questionnaire. The questionnaire has been back-translated into German by an independent translator for validation. Self-reporting is subjected to recall bias; however, the use of a translated questionnaire is more inclusive for immigrant populations. | |
| 1. Representativeness – Score: 0 | |
| The sample size is relatively small (560) and homogeneous in terms of living conditions. Participants are asylum seekers living in the same shared accommodation, which may limit generalisability of results. Gender distribution (M>F) is representative of the asylum seeker population. | |
| 1. Exposure: immigration — Score: -1 | |
| No information is provided on how immigration status is defined and measured, and no other immigration factors are measured and accounted for, limiting the impact of the study. | |
| 1. Outcome: chronic pain — Score: -1 | |
| No information is provided on how chronic pain is defined and measured, and no other pain characteristics are investigated, limiting the rigour of the study. | |
| 1. Study design — Score: -1 | |
| This study would benefit by accounting for demographic and immigration variables and comparing findings to an unexposed group. | |
| Study | |
| Chronic pain in multi-traumatized outpatients with a refugee background resettled in Norway: a cross-sectional study (Teodorescu et al 2015) – Total score: -1 | |
| 1. Data collection – Score: 0 | |
| This cross-sectional study investigates the prevalence of chronic pain in PTSD patients with a refugee background. Data are collected from a structured clinical interview for assessing PTSD and chronic pain and various immigration factors are reported using a self-report questionnaire. A structured interview is a more reliable way than self-reporting health outcomes, however, a clinical assessment and diagnosis would have been an even more robust method. | |
| 1. Representativeness – Score: -1 | |
| The sample is poorly representative of both the general and immigration population. The sample size is very small (61 participants), and only patients with a PTSD diagnosis, with a permanent residence permit, and who are proficient in both written and oral Norwegian are included in the study. Therefore, prevalence rates may be biased and should be interpreted with caution. | |
| 1. Exposure: immigration — Score: 0 | |
| Refugee background is defined and measured by a single self-reported question: ‘Which is your country of origin?’. This has the advantage of being simple and straightforward. A strength of the study is that it assesses and accounts for integration in Norway and in the ethnic community. However, other important migration factors, such as length of stay, are not investigated. | |
| 1. Outcome: chronic pain – Score: 1 | |
| Chronic pain is defined as a long-lasting pain stretching over many years and was assessed using a single item from The Structured Interview for Disorders of Extreme Stress (SIDES)*:* ‘I suffer from chronic pain’. This is a clear and straightforward method, but vague. Location and severity are also reported using the SIDES scale. A medical assessment and diagnosis would be a more reliable method of reporting chronic pain. | |
| 1. Study design – Score: -1 | |
| The main limitations of this study are its small sample size, low representativeness, that it did not assess and control for migration variables, and that there was no comparison group. | |
| Study | |
| The association between health and sickness absence among Danish and non-Western immigrant cleaners in Denmark (Carneiro et al 2013) – Total score: 0 | |
| 1. Data collection – Score: -1 | |
| This cross-sectional study investigates the association between health and sickness absence among Danish and non-Western immigrant cleaners in Denmark. Data are collected using self-reported questionnaires, which is subjected to recall bias. | |
| 1. Representativeness — Score: 0 | |
| The sample size is relatively small (n = 276), reducing the generalisability of results. The gender (F>M) and age distribution (Danes 51% and Non-Westerners 75%: 30–50 years old) reflect that of the occupational population of cleaners. The immigrant group consists of many different nationalities, making findings more challenging to interpret but may, on the other hand, be more representative of the immigrant communities in Denmark and Europe. | |
| 1. Exposure: Immigration – Score: 0 | |
| Immigration status is clearly defined based on country of birth and only first-generation immigrants are classified as immigrants. However, no information is provided on how the country of birth is reported, and no other immigration factors are investigated. | |
| 1. Outcome: Chronic pain – Score: 0 | |
| Total body pain is calculated using the Standardised Nordic Questionnaire, and chronic pain is defined as pain lasting over 30 days in the last year. This is the shortest duration definition of chronic pain among the studies included in this review and potentially poses validity issues. Location, intensity, and other pain variables are not investigated. | |
| 1. Study design – Score: 1 | |
| One of the strengths of this study is that it compares results with a similar unexposed group (Danish cleaners). Another advantage is controlling for age, gender, employment status, smoking, and BMI, however, it does control not for immigration variables. | |
| Study | |
| The burden of chronic pain: a cross-sectional survey focussing on diseases, immigration, and opioid use (Kurita et al 2012) – Total score: 4 | |
| 1. Data collection — Score: 0 | |
| The aim of this cross-sectional study is to estimate the prevalence of chronic pain in the adult Danish population and to analyse associated factors such as immigration. Demographic data are obtained from the Danish Civil Registration System, which minimises bias. Data on chronic pain are collected from a self-reported questionnaire in Danish, which is subject to recall bias and potentially excludes immigrants who are not fluent in written Danish. | |
| 1. Representativeness – Score: 1 | |
| The sample size is relatively large (14,925) and representative of the national demographic characteristics with an adequate response rate (60.7%). The immigrant group consists of many different nationalities, and therefore is less homogeneous but more representative of immigrant groups in Denmark and Europe. | |
| 1. Exposure: Immigration – Score: 1 | |
| Immigration is clearly defined and measured based on data from the Danish Civil Registration System, but no other immigration characteristics and acculturation are measured and accounted for. | |
| 1. Outcome: Chronic pain — Score: 1 | |
| Chronic pain is clearly defined and self-reported by a single question: ‘Do you have chronic/long-lasting pain lasting more than 6 months?’. This has the advantage of being clear and is the longest duration definition reported among the studies included in this review. In addition, location and intensity of pain are also measured and investigated for. A medical assessment and diagnosis would be a more robust method of reporting chronic pain. | |
| 1. Study design – Score: 1 |  |
| Advantages of this study include the large sample, the unexposed comparison group and that is controls for age, gender, education, BMI, and cohabitation status. Findings would be enriched if immigration factors were investigated. | |
