# Supplementary material for: Exploring the burden, prevalence and associated factors of chronic musculoskeletal pain in migrants from North Africa and Middle East living in Europe: a scoping review
Source: BMC Public Health. 2024 Mar 12;24:769. doi: 10.1186/s12889-023-17542-2 (PMC10935970; doi:10.1186/s12889-023-17542-2)
Supplement: Supplementary file 3 — Additional file 3. Study characteristics and results. [file 12889_2023_17542_MOESM3_ESM.docx]

**Additional file 3. Study characteristics and results**

| Author | Country | Type of study | Population | Results |
| --- | --- | --- | --- | --- |
| Keshk 2021 | unknown transit | retrospective cohort | 2512 victims of torture patients in a *Médecins sans frontiers* clinic | Prevalence of chronic pain: |
|  |  |  |  | 46% of the 1629 patients who report physical symptoms |
|  |  |  | **F:** ? | 29.90% of all patients attending the clinic |
|  |  |  | **Age:** 20–45 years: 82.3% | Physiotherapy: |
|  |  |  | **Country of origin:** ? | 37% of all cases were referred to physiotherapy |
|  |  |  | **Migration status:** in transit | of those 19.7% improved |
|  |  |  |  | 5 sessions were needed for a successful outcome in physiotherapy |
| Strømme 2020 | Norway | prospective cohort | 353 refugees, relocating from Lebanon to Norway | Prevalence of chronic pain: |
|  |  |  |  | baseline: 30% |
|  |  |  | **F:** 51.3 % | follow-up: 28% |
|  |  |  | **Median age:** 34 years | Change of chronic pain from Lebanon to Norway: |
|  |  |  | **Country of origin:** Syria | OR: 0.92 [0.68–1.23] |
|  |  |  | **Migration status:** in transit and early post-migration | not significant |
|  |  |  |  |  |
| Strømme 2020 | Norway | cross-sectional | 827 refugees | Prevalence of chronic pain: |
|  |  |  | Of which in Lebanon: 506 | total sample: 30% [27–33] |
|  |  |  | Of which in Norway: 321 | Lebanon: 31% [27–35] |
|  |  |  | **F:** 41% | Norway 37% [31–43] |
|  |  |  | **Median age:** 33 years | the most prevalent condition reported |
|  |  |  | **Country of origin:** Syria | Gender differences chronic pain: |
|  |  |  | **Migration status:** in transit and early post-migration | Lebanon: Syrian women 22% [17–28] vs Syrian men 15% [11–20] |
|  |  |  | **Migration status:** in transit and early post-migration | Norway: Syrian women 32% [22–44] vs Syrian men17% [12–23] |
|  |  |  |  | Gender differences painkiller usage: |
|  |  |  |  | Lebanon: Syrian women 14% [10 - 19] vs Syrian men 4% [2 -7] |
|  |  |  |  | Norway: Syrian women 13% [6–22] vs Syrian men 9% [5–14] |
|  |  |  |  | Chronic pain was associated with: |
|  |  |  |  | gender, age, education, country, time in transit, and traumatic experience |

| Author | Country | Type of study | Population | Results |
| --- | --- | --- | --- | --- |
| Waxenegger 2017 | Austria | cross-sectional | Total participants: 15,748 | Mean musculoskeletal pain (out of four) |
|  |  |  | Austrians: 14,268 (90%) | Austrians: 0.60 SD: 0.93 |
|  |  |  | Immigrants very high HDI: 542 | Immigrants very high HDI: 0.55 SD: 0.89 |
|  |  |  | Immigrants high HDI: 363 | Immigrants high HDI: 0.75 SD: 1.09 |
|  |  |  | Immigrants low HDI: 575 | Immigrants low HDI: 0.65 SD: 0.95 |
|  |  |  | **F:** | Musculoskeletal pain adjusted for age (regression coefficient) |
|  |  |  | Total participants: 55.7% | Immigrant men very high HDI significantly worse than men WMB: -0.115 [-.225–0.363] p=0.001–0.009 |
|  |  |  | Austrians: 55.2% | Immigrant men low HDI significantly worse than men WMB: 0.107 [0.006–0.208] p = 0.001–0.009 |
|  |  |  | Immigrants very high HDI: 61.3% | Immigrant women very high HDI significantly worse than women WMB: -0.052 [-0.103–0.001] p=0.001-0.009 |
|  |  |  | Immigrants high HDI: 65.8% |  |
|  |  |  | Immigrants low HDI: 56.7% | Immigrant low HDI significantly worse than women WMB: 0.060 [0.008–0.112] p=0.001–0.009 |
|  |  |  | **Age:** | Musculoskeletal pain adjusted for age, SES and health-related habits (regression coefficient) |
|  |  |  | Total participants: | Immigrant women low HDI significantly worse than women WMB: 0.162 [0.056–0269] |
|  |  |  | 26.3%: 30–44 yrs, 34.1%: 45–59 yrs | Physical Quality of Life adjusted for age, SES and health-related habits (regression coefficient) |
|  |  |  | Austrians: | Immigrant women high HDI significantly worse than women WMB: -0.375 [-0.694–-0.056] |
|  |  |  | 25.5%: 30–44 yrs, 35%: 45–59 yrs | Immigrant women low HDI significantly worse than women WMB: -0.541 [-0.829–-0.262] |
|  |  |  | Immigrants very high HDI: | Psychological Quality of Life adjusted for age, SES and health-related habits (regression coefficient) |
|  |  |  | 30.1%: 30–44 yrs, 30.6%: 45–59 yrs | Immigrant men high HDI significantly worse than men WMB: -0.483 [-0.859–-0.107] |
|  |  |  | Immigrants high HDI: | Immigrant men low HDI significantly worse than men WMB: -0.280 [-0.552–-0.008] |
|  |  |  | 31.7%: 30–44 yrs, 25.3%: 45–59 yrs | Subjective health adjusted for age, SES and health-related habits (regression coefficient) |
|  |  |  | Immigrants low HDI: | Immigrant women high HDI significantly worse than women WMB: 0.154 [0.069–0.240] |
|  |  |  | 28.9%: 15–29 yrs, 40.2%: 30–44 yrs | BMI adjusted for age, SES and health-related habits (regression coefficient) |
|  |  |  | **Country of origin:** | Immigrant women high HDI significantly worse than women WMB: 0.486 [-0.981–0.009] |
|  |  |  | Asia & Africa: 8.8% | Immigrant men very high HDI significantly worse than men WMB: -0.583 [-1.111–-0.055] |
|  |  |  | **Immigration status:** | Immigrant men high HDI significantly worse than men WMB: 0.740 [0.059–1.422] |
|  |  |  | Migration background: foreign-born or both parents foreign-born | Immigrant men low HDI significantly worse than men WMB: 0.851 [0.357–1.345] |

| Author | Country | Type of study | Population | Results |
| --- | --- | --- | --- | --- |
| Rosenkrands 2020 | Denmark | cross-sectional | 408 immigrant patients with complex needs | prevalence of musculoskeletal pain: |
|  |  |  |  | most prevalent symptom (percentage not reported) |
|  |  |  | **F:** 83% | prevalence of pain locations: |
|  |  |  | **Mean age:** 49 years | arms/hands/legs/knees/hips: 87% (F: 87%, M: 83%) |
|  |  |  | **Country of origin:** 43 nationalities, Middle East 71%, Asia 2%, Africa 20% | shoulder/neck: 83% (F: 86%, M: 71%) |
|  |  |  |  | upper/lower back: 83% (F: 85%, M: 75%) |
|  |  |  |  | headache: 82% (F: 83%, M: 75%) |
|  |  |  | **Migration status:** 63% > 20 years residence permit |  |
| Dragioti 2020 | Sweden | prospective cohort | 15,563 participants  90% Swedish-born  10% immigrants | Prevalence of chronic pain: |
|  |  |  |  | foreign-born: 45% |
|  |  |  |  | Swedish-born: 39% |
|  |  |  | **F:**  total sample 54%,  Swedish-born: 54%  foreign-born: 55% | significant difference: p < 0.001 |
|  |  |  |  | Prevalence of chronic widespread pain: |
|  |  |  |  | foreign-born: 11% |
|  |  |  |  | Swedish-born: 8% |
|  |  |  | **Mean age:**  total sample 51.6,  Swedish-born: 51.9  foreign-born: 48.9 | significant difference: p < 0.001 |
|  |  |  |  | Prevalence of severe chronic pain: |
|  |  |  |  | foreign-born: 32% |
|  |  |  |  | Swedish-born: 20% |
|  |  |  | **Country of origin:**  Africa 72%  Asia and Oceania: 30%  Europe: 40%  Nordic countries: 17%  North America: 3%  South America: 5% | significant difference: p < 0.001 |
|  |  |  |  | Risk of pain outcomes in immigrants: |
|  |  |  |  | chronic pain OR: 1.18 [1.04–1.33] |
|  |  |  |  | chronic widespread pain OR: 1.39 [1.15–1.69] |
|  |  |  |  | severe chronic pain OR: 1.51 [1.23–1.87] |
|  |  |  |  | Baseline variables with direct significant increased effect on chronic pain at follow-up: |
|  |  |  |  | age (bstd = 0.19, p < 0.001) |
|  |  |  | **Migration status:** ? | immigration status (bstd = 0.02, p = 0.01) |
|  |  |  |  | mood (anxiety and depression) (bstd =0.02, p = 0.01) |
|  |  |  |  | Baseline variables with direct significant decreased effect on chronic pain at follow-up: |
|  |  |  |  | university education (bstd = − 0.04, p < 0.001) |

| Author | Country | Type of study | Population | Results |
| --- | --- | --- | --- | --- |
| Pfortmueller 2016 | Switzerland | cross-sectional | 880 refugees or asylum seekers patients to emergency department | prevalence of chronic MSK pain: |
|  |  |  |  | 12.30% |
|  |  |  | **F:** 29.6% | second most common chronic disorder |
|  |  |  | **Mean age:** 34 years |  |
|  |  |  | **Country of origin:** Middle East (25.2% Syrian) |  |
|  |  |  | **Migration status:** ? |  |
| Führer 2016 | Germany | cross-sectional | 214 asylum seekers | prevalence of pain: |
|  |  |  | **F:** 11% | 37.80% |
|  |  |  | **Mean age:** ? | of those: 44% reported to feel pain often, most of the time or all the time |
|  |  |  | **Country of origin:** ? | prevalence of chronic pain (in total): |
|  |  |  | **Status of migration:** ? | 16.60% |
| Teodorescu 2015 | Norway | cross-sectional | 61 psychiatric patients with refugee background | prevalence of chronic pain (at least one location): 98% |
|  |  |  |  | mean pain locations per patient: 4.6 |
|  |  |  | **F:** 41% | prevalence of chronic pain at clinical levels: 66% |
|  |  |  | **Mean age:** 41.7 years | most prevalent chronic pain locations: |
|  |  |  | **Country of origin:** 21 countries from four continents | head 80% |
|  |  |  |  | chest 74% |
|  |  |  | **Status of migration:** ? | arms/legs 66% |
|  |  |  |  | back 62% |
|  |  |  |  | gender and chronic pain: |
|  |  |  |  | **chronic pain locations** |
|  |  |  |  | women μ, SD = 5.28, 1.0 |
|  |  |  |  | men μ, SD = 4.08, 2.13 |
|  |  |  |  | significant difference p = 0.027 |
|  |  |  |  | no other significant difference between genders |
|  |  |  |  | comorbidity: |
|  |  |  |  | PTSD and chronic pain: 57% |
|  |  |  |  | chronic pain in PTSD patients: 88% |
|  |  |  |  | PTSD in patients with chronic pain at clinical levels: 70% |
|  |  |  |  | chronic pain patients had more symptoms of PTSD, depression and anxiety |
|  |  |  |  | associations: |
|  |  |  |  | no significant difference between chronic pain and no chronic pain for: age, gender, marital status, ethnicity, living conditions (alone or with others), employment, having friends, proficiency in Norwegian, integration in Norway, integration in ethnic community, years in Norway, education |

| Author | Country | Type of study | Population | Results |
| --- | --- | --- | --- | --- |
| Carneiro 2012 | Denmark | cross-sectional | 276 cleaners | total body pain: |
|  |  |  | **Danish**: 47% **F:** 87.5%  **Mean age:** 47.6 | Non-Western cleaners: μ, SD = 10.1, 6.9 |
|  |  |  |  | Danish cleaners: μ, SD = 8.0, 5.5 |
|  |  |  |  | Significant difference p = 0.007 |
|  |  |  | **Non-Western immigrants:** 48%  **F:** 74.2%  **Mean age:** 42.2% | total body pain & sickness absence: |
|  |  |  |  | significantly associated in Non-Western cleaners only (adjusted OR = 1.14 [1.04–1.26]) |
|  |  |  |  |  |
|  |  |  | **Country of origin:** Turkey 24% Macedonia 12% Thailand 10% Pakistan 9% |  |
|  |  |  | **Migration status:** foreign-born |  |
| Kurita 2012 | Denmark | cross-sectional | 14925 participants | Prevalence of chronic pain: |
|  |  |  | **F:** 64.9 % | Non-Western immigrants: 32.8% |
|  |  |  | **Mean age:** ? | Danes: 26.4% |
|  |  |  | **Country of origin:**  Danish 63.2%  Western: 42.1%  Non-Western: 33.7% | Other Westerners: 27.1% |
|  |  |  |  | Odds of reporting chronic pain: |
|  |  |  |  | 1.68 [1.41 - 1.98] higher in immigrants than Danes |
|  |  |  |  | Mean number of pain locations: |
|  |  |  | **Migration status**: ? | Non-Western immigrants: 3.1 |
|  |  |  |  | Danes: 2.1 |
|  |  |  |  | Other Westerners: 2.0 |
|  |  |  |  | Prevalence of widespread pain: |
|  |  |  |  | Non-Western immigrants: 10.3% |
|  |  |  |  | Danes: 4.6% |
|  |  |  |  | Other Westerners: 4.1% |
|  |  |  |  | Mean pain intensity (out of 10) |
|  |  |  |  | Non-Western immigrants: 6.2 |
|  |  |  |  | Danes: 4.8 |
|  |  |  |  | Other Westerners: 4.3 |

F: female, OR: odds ratio, [ ]: standard deviation, HDI: Human Development Index, WMB: without migration background M: male, SES: socioeconomic status, bstd: standardised regression coefficient, μ: mean, SD: standard deviation, PTSD: post-traumatic stress disorder
